# Giant photoluminescence enhancement in MoSe_2_ monolayers treated with oleic acid ligands†

**DOI:** 10.1039/d0na01014f

**Published:** 2021-06-12

**Authors:** Arelo O. A. Tanoh, Jack Alexander-Webber, Ye Fan, Nicholas Gauriot, James Xiao, Raj Pandya, Zhaojun Li, Stephan Hofmann, Akshay Rao

**Affiliations:** Cavendish Laboratory, Cambridge JJ Thomson Avenue CB3 0HE Cambridge UK ar525@cam.ac.uk; Cambridge Graphene Centre, University of Cambridge 9 JJ Thomson Avenue CB3 0FA Cambridge UK; Department of Engineering, University of Cambridge JJ Thomson Avenue CB3 0FA Cambridge UK

## Abstract

The inherently low photoluminescence (PL) yields in the as prepared transition metal dichalcogenide (TMD) monolayers are broadly accepted to be the result of atomic vacancies (*i.e.*, defects) and uncontrolled doping, which give rise to non-radiative exciton decay pathways. To date, a number of chemical passivation schemes have been successfully developed to improve PL in sulphur based TMDs *i.e.*, molybdenum disulphide (MoS_2_) and tungsten disulphide (WS_2_) monolayers. Studies on solution based chemical passivation schemes for improving PL yields in selenium (Se) based TMDs are however lacking in comparison. Here, we demonstrate that treatment with oleic acid (OA) provides a simple wet chemical passivation method for monolayer MoSe_2_, enhancing PL yields by an average of 58-fold, while also improving spectral uniformity across the material and reducing the emission linewidth. Excitation intensity dependent PL reveals trap-free PL dynamics dominated by neutral exciton recombination. Time-resolved PL (TRPL) studies reveal significantly increased PL lifetimes, with pump intensity dependent TRPL measurements also confirming trap free PL dynamics in OA treated MoSe_2_. Field effect transistors show reduced charge trap density and improved on–off ratios after treatment with OA. These results indicate defect passivation by OA, which we hypothesise as ligands passivating chalcogen defects through oleate coordination to Mo dangling bonds. Importantly, this work combined with our previous study on OA treated WS_2_, verifies OA treatment as a simple solution-based chemical passivation protocol for improving PL yields and electronic characteristics in both selenide and sulphide TMDs – a property that has not been reported previously for other solution-based passivation schemes.

## Abstract 2

Following our previous work on WS_2_ surface treatment with oleic acid (OA), we show that OA also greatly improves the optoelectronic characteristics of MoSe_2_. Optical and electronic measurements point to selenide vacancy passivation by OA ligands.

## Graphical abstract

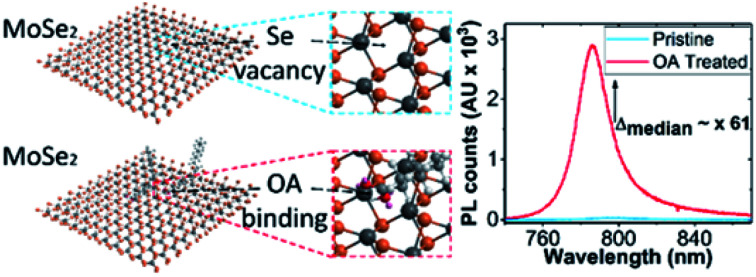

Two-dimensional (2D) (or monolayer) transition metal dichalcogenides (TMDs) continue to attract wide-spread research interest due to their intriguing optical and electronic properties.^2–4^ A few to hundreds of micron sized monolayers can be isolated from their layered bulk counterparts by overcoming the interlayer van der Waals interaction *via* various layer by layer exfoliation methods. These include dry mechanical cleavage,^4,5^ metal assisted exfoliation^6^ and liquid phase exfoliation (LPE).^7^ There are also continuous efforts to grow wafer-scale crystalline monolayers *via* epitaxial growth methods such as chemical vapour deposition (CVD).^8^ A number of TMDs transition from an indirect optical gap in the bulk crystal to a direct optical gap as a monolayer.^9^ The direct optical gap, high absorption^10^ and potentially high charge carrier mobilities of a number of monolayer TMDs have spurred research into their application in optoelectronic devices namely photodetectors, light emitting diodes (LEDs),^11^ field effect transistors (FETs)^3^ and on-chip single photon quantum emitters.^12^ Moreover, the massively reduced dielectric screening gives rise to tightly bound excitons^2,9^ even at room temperature, thus providing a convenient means to study the many-body exciton–exciton and exciton–charge interactions that give rise to a multitude of exotic neutral excitons^13^ and charged excitons.^14,15^

Although monolayer TMDs hold great promise for future optoelectronic applications, the as-prepared monolayers tend to exhibit low photoluminescence quantum efficiency (PLQE).^4,11^ The persistence of non-radiative pathways in pristine monolayers has been mainly attributed to chalcogen (*i.e.,* S and Se) vacancies,^16,17^ atomic substitutions^18,19^ and trion formation.^16,20^ Chalcogen vacancies (CVs) and atomic substitutions are examples of structural defects which come under the category of point defects.^21^ CVs in particular are predicted to be the prevalent form of structural defect in newly fabricated monolayers due to their low formation energy.^21–23^ CVs are known to act as charge traps, where excitons quench non-radiatively due to charge separation, or bind with trapped charges to form trions, which have low radiative efficiency, resulting in an overall reduction in PLQE.^16,20^ CVs also trap mobile charge carriers, hampering electronic performance. Other structural defects include grain boundaries, which induce local strain, altering the local electronic structure in polycrystalline large area CVD prepared monolayers. In the absence of grain boundaries, as in the single crystal monolayers studied in this work, chalcogen defects are considered to be the dominant structural defect on account of their low formation energy.^21–23^ Externally induced sources of disorder also undermine the material performance of monolayer TMDs. External sources of disorder originate from the underlying substrate and ambient adsorbates. Substrate induced disorder includes surface strain and unintentional doping. These external perturbations cause charge scattering, charge trapping and local band structure modifications, which hamper electron mobilities and quench monolayer PL, respectively.^21^ Charged impurities and substrate doping introduce free charge carriers, which can contribute to the conversion of bright neutral excitons to trions.^16,20^

Methods to improve material performance broadly take two routes: encapsulation or chemical passivation. Encapsulation utilizes the atomically flat dielectric properties of hexagonal boron nitride (hBN), using it as an encapsulation medium^24,25^ or sub-layer^26^ that isolates TMD monolayers from doping and disorder imposed by common substrate materials.^21^ This preserves their intrinsic properties and improves the overall optical quality as indicated by spatially homogeneous narrow linewidths in the PL spectra. Encapsulation with hBN has been shown to suppress exciton–exciton annihilation in monolayer tungsten disulphide (WS_2_), improving PL, however, at high excitation intensities.^27^ Large PL enhancement at low excitation density has not been demonstrated with hBN encapsulation alone.

On the other hand, recently, a number of successful chemical passivation schemes have been devised to enhance the PLQE of sulphur based TMDs, namely molybdenum disulphide (MoS_2_) and tungsten disulphide (WS_2_). Such methods involve the use of p-doping agents such as 2,3,5,6-tetrafluoro-7,7,8,8-tetracyanoquinodimethane (F4TCNQ),^28,29^ hydrogen peroxide,^30^ and sulphuric acid,^31^ or deposition of a monolayer titanyl phthalocyanine (TiOPc) charge transfer interface.^32^ These techniques aim to withdraw electrons to suppress the formation of low PLQE trions, promoting dominant neutral exciton recombination. One of the most successful of these chemical treatments has been the use of the non-oxidizing ‘super acid’ bis(trifluoromethane)sulfonimide (TFSI)^16,33,34^ to treat MoS_2_ and WS_2_, leading to large increases in PL. It has been suggested that TFSI acts as a strong electron withdrawing (p-doping) species *via* comparative studies with gated n-type MoS_2_ and WS_2_ monolayers, whereby applying a negative bias suppresses non-radiative pathways *via* trion formation, leaving dominant neutral exciton recombination and similar PL dynamics to TFSI treated monolayers.^20^ However, PL dynamics in TFSI treated MoS_2_ and WS_2_ have been shown to be trap-limited,^1,35^ and it has been recently shown that this is due to the presence of sulphur vacancies which remain unpassivated even with the TFSI treatment.^36^

Recently, the authors of this study demonstrated a 26 and 20 fold increase in WS_2_ PL and electron mobilities, respectively, *via* surface treatment with Oleic Acid (OA) ligands, outperforming treatment with TFSI.^1^ The OA treatment results in high spectral uniformity with non-trap limited PL dynamics compared with TFSI treated monolayers, which indicate defect passivation by OA ligands. In support of this, electrically gated monolayers treated with OA show increased field effect mobilities with reduced charge trap density and no additional doping in comparison to their untreated or ‘pristine’ form. The study also revealed bright trion PL evolution in OA treated WS_2_ at high excitation densities due to the binding between untrapped excitons and local n-type charges. This strong trion evolution has potential applications in quantum information processing. The authors suggested defect passivation *via* dative covalent bonding between the oleate group on the OA ligand and metal atoms at the chalcogen vacancy, which prevents defect/trap assisted non-radiative exciton decay and promotes direct band-edge recombination – thus improving PL yields in a manner akin to defect passivation by OA in colloidal nanocrystals.

In contrast to the range of chemical treatments for sulphur based TMDs, there has been little success in developing treatments for selenium based TMDs *i.e.* molybdenum diselenide (MoSe_2_) and tungsten diselenide (WSe_2_).^33^ For instance, Amani *et al.*^33^ showed that TFSI quenches PL in both these materials instead of enhancing it, which is in stark contrast to sulphide TMDs which respond well to TFSI treatment, yielding monolayers with bright PL. The authors attributed this outcome to differences in the nature of defects between selenide and sulphide TMDs, with no further explanation. So far, the underlying reasons for this remain unclear. Han *et al.*^37^ achieved a 30-fold enhancement of defect rich CVD MoSe_2_ PL at room temperature *via* exposure to hydrobromic acid (HBr) vapour. The authors attributed this outcome to p-doping with HBr combined with structural repair of chalcogen vacancies. Structural repair occurs *via* the replacement of oxygen substitutions by bromine (Br) ions at selenium (Se) vacancies which acts to suppress trapped exciton states, thus eliminating non-radiative pathways. Recently, high PLQE of the as-prepared CVD WSe_2_ has been demonstrated *via* solvent evaporation-mediated decoupling (SEMD),^38^ whereby the solvent evaporation process assists in the separation of the as-grown synthetic monolayers from the underlying substrate. This serves as an alternative to polymer assisted transfer methods, which involve the use of harsh chemicals *e.g.*, hydrofluoric acid (HF). The drastic improvement in optical quality compared to that obtained by the standard CVD monolayer transfer technique is considered to be related to overcoming substrate induced mechanical strain, which can introduce band structure modifications that reduce PL.^38,39^ These methods however, do not provide the ease of processing that simple solution based chemical approaches do and rely on specific growth conditions, restricting their general purpose application.

Here, we demonstrate that oleic acid (OA) treatment of MoSe_2_ monolayers results in greatly enhanced neutral exciton PL, as well as trap-free PL dynamics. In addition, OA treated MoSe_2_ field effect transistors (FETs) exhibit marked improvement in transfer characteristics. The reduced subthreshold swing (SS) indicated reduced charge trap density and hence improved current on/off ratios. In combination with our previous study on OA treated WS_2_,^1^ these results highlight OA treatment as a solution-based passivation protocol applicable to both selenide and sulphide TMDs, which has not been previously reported in the wider literature. This work therefore underlines OA treatment as a simple, versatile post-fabrication solution based chemical passivation protocol for both selenide and sulphide TMDs, which is devoid of harsh chemicals and does not require specific growth conditions.

## Experimental

### Monolayer exfoliation

Prior to exfoliation, substrates were solvent processed *via* sonication in acetone and isopropyl alcohol (IPA) for ∼15 min and treated in O_2_ plasma to remove adsorbants. Silicon–silicon dioxide (Si–SiO_2_) substrates with a 90 nm oxide layer were used for steady state PL measurements, while thin (∼170 μm) 22 mm × 22 mm glass cover slides were used for steady state excitation intensity dependent PL, TRPL and Raman microscopy.

Large area MoSe_2_ monolayers were prepared *via* gold-mediated exfoliation.^6^ The bulk crystal purchased from 2D Semiconductors was exfoliated manually onto low density clean-room tape prior to depositing a thin gold layer (∼100–150 nm) *via* thermal evaporation under vacuum conditions. Following gold evaporation, thermal release tape was adhered to the gold coated MoSe_2_ exfoliate, whereupon the cleanroom tape was peeled off, leaving the top-most layer of MoSe_2_ attached to the gold on thermal tape. The thermal tape was then stuck onto the freshly plasma treated target substrate and heated on a hot plate up to 125 °C, peeling the thermal tape and leaving the TMD monolayers sandwiched between the gold and substrate. Excess gold was removed by immersing in potassium iodide (KI_2_) and iodine (I_2_), a standard gold etchant (Sigma Aldrich). The substrate was gently swirled in the etchant for 5 minutes prior to rinsing in deionised water followed by 10 minute sonication in acetone and 5 minute rinse in isopropyl alcohol (IPA). Samples were dried with a nitrogen (N_2_) gun. Monolayers were initially identified *via* the optical contrast method^40^ and PL microscopy.

### Oleic acid treatment

In a nitrogen glovebox, pure degassed OA (Sigma Aldrich) was drop cast onto the exfoliated monolayer TMD samples – enough to thinly coat the area of the exfoliated material on the substrate. The samples were then placed on a hot plate set to 25 °C for 12 hours overnight. After treatment, the samples were rinsed in anhydrous toluene and blow dried with a N_2_ gun. Using PL spectroscopy, as shown in ESI Fig. 1,† we show that toluene marginally improves PL in WS_2_. This is attributed to the removal of impurities that otherwise quench PL. We predict a similar effect with MoSe_2_. Sample optical micrographs of MoSe_2_ monolayers before and after OA treatment are provided in ESI Fig. 2.†

### Steady state PL microscopy

PL spectroscopy was performed on a Renishaw Invia confocal setup equipped with a motorized piezo stage, using an air-cooled Ar-ion (argon ion) 514.5 nm continuous wave (CW) laser *via* 50× objective (NA = 0.75). Signals were collected in reflection *via* a notch filter. The diffraction limited beam spot size was estimated to be 0.84 μm. The PL signal was dispersed *via* a 600 l mm^−1^ grating prior to detection with an inbuilt CCD detector. The laser power was measured directly *via* a 5× objective with a Thorlabs S130C photodiode and PM100D power meter.

PL maps were generated from multiple MoSe_2_ monolayers before and after OA treatment. Maps were generated with 1 μm resolution and 0.5 s integration time at 0.7 μW. Steady state intensity dependent PL series measurements were performed on the MoSe_2_ monolayer on a glass slide. Care was taken to measure PL in the same location before and after treatment. The spatial *x*, *y* and *z* focal planes of the measurement site and photodiode were recorded to accurately switch between the photodiode and monolayer location for each PL measurement using the WIRE software piezo stage control interface. PL signals were scaled up to 500 s integration time, which was used for the lowest excitation intensity measurement. Dark counts were recorded with the same integration time used for each PL measurement in the series. Dark counts were scaled and subtracted from the raw PL data accordingly.

**Fig. 1:**
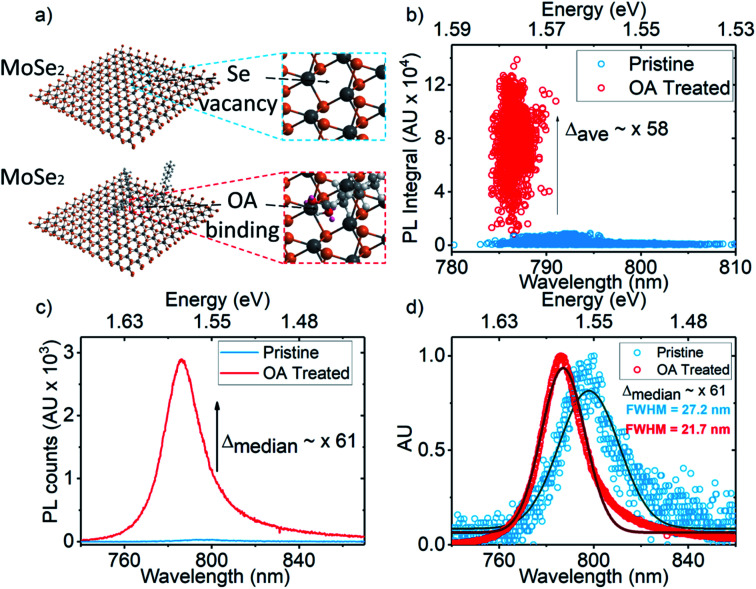
(a) Illustration of OA ligand coordination to a selenium (Se) vacancy; (b) PL enhancement scatter plot showing untreated monolayer PL integrals (blue) and OA treated monolayer PL integrals (red); (c) raw PL spectra for points that represent the median PL integrals before (blue) and after OA treatment (red) on an exemplary monolayer; (d) normalised median spectra (circles) from (c), with single Gaussian peaks (solid lines) fitted to estimate the change in FWHM from untreated (blue) to OA treated (red) cases.

A single Gaussian fit from the standard peak fit library in Origin lab was used, as in Fig. 1d, to estimate changes in FWHM between untreated and OA treated MoSe_2_ PL signals. Exciton species in Fig. 3a were deconvoluted from OA treated MoSe_2_ PL signals with dark counts subtracted using a procedure written in Matlab, which incorporates the ‘gauss2’ two Gaussian model fit. Further information on the Gaussian model is available on the *Mathworks* website.

### Raman microscopy

MoSe_2_ monolayers on thin glass cover slides were characterized *via* Raman spectroscopy using a Renishaw Invia confocal setup. Excitation was provided using a 530 nm CW laser *via* 50× objective (NA = 0.75), producing an estimated diffraction limited beam spot size of 0.86 μm. The Raman signal was collected in reflection *via* a notch filter and dispersed with a 1800 l mm^−1^ grating prior to detection with an inbuilt CCD camera.

### Time resolved PL microscopy

TRPL measurements were performed using a custom-built inverted PL microscope setup equipped with a motorized piezo stage. Excitation was provided by a pulsed super continuum white light source (Fianium Whitelase) filtered *via* a Bentham TMc 300 monochromator. TRPL excitation was acquired using a 550 nm laser *via* a 60× oil objective, producing a 10 μm diameter confocal laser spot on the sample. The laser spot size was measured using the image created on an EMCCD camera (Photometrics QuantEM™ 512SC). The laser repetition rate was set to 5 MHz with 11.4 ps resolution to obtain PL decay data. The MoSe_2_ PL was collected using a MPD visible single photon avalanche diode (Vis-SPAD) *via* 750 nm long pass and 900 nm short pass filters, completely filtering out laser excitation and allowing collection of MoSe_2_ PL only. Further precaution was taken to remove any long wave component of the excitation line using a 650 nm short pass filter. All signals were scaled up to 3000 s, which was used for the lowest excitation intensity measurement. The laser power was measured with the excitation line using a Thorlabs S130C photodiode and PM100D power meter. The laser excitation power was regulated using a series of neutral density filters. The instrument response function was measured with a blank glass cover slide, as used for the sample. Decay rates were fitted using a model developed in Origin, which consists of a Gaussian (as the IRF) convoluted with a double exponential decay.

### Transistor preparation and characterisation

After exfoliation and transfer onto Si–SiO_2_ (90 nm), isolated monolayer MoSe_2_ flakes were identified and electrodes with a typical channel length of 4 μm were patterned using e-beam lithography and thermal evaporation of Pd : Au (20 nm : 80 nm), followed by lift-off in acetone. Transfer characteristics were measured using a Keithley 4200 SCS connected to a probe station. The global back-gate was swept from negative to positive voltages and the current was measured under a source-drain bias of 5 V.

### Nanocrystal graphics

Nanocrystal graphics in Fig. 1a were developed in VESTA software^41^ and parsed into ChemDraw3D (PerkinElmer) for rendering.

## Results & discussion

Fig. 1a shows a cartoon illustration of OA ligand coordination to a selenium (Se) vacancy. Fig. 1b shows the scatter plot of the spectral position of peak emission and PL integrals extracted from PL maps of multiple MoSe_2_ monolayers on Si–SiO_2_ substrates before and after OA treatment. Maps were measured at 126 W cm^−2^. Fig. 1c shows the PL spectra for points on an exemplary monolayer that correspond to the median PL enhancement, *Δ*_median_, where *Δ* = PL_after treatment_/PL_before treatment_. Table 1 shows the statistical information derived from Fig. 1b, namely: average PL enhancement across the monolayers (*Δ*_ave_); standard deviation in the PL integral (*σ*_PL_); average emission peak wavelength (*λ*_ave_); and standard deviation in the peak wavelength (*σ*_*λ*_). The untreated case is indicated by (*). Fig. 1d shows the normalised spectra of Fig. 1c, with single Gaussian peaks fitted to estimate the change in spectral linewidth between untreated (blue) and treated (red) cases.

**Table 1:** PL enhancement statistics derived from PL maps of MoSe_2_ monolayers. Characteristics prior to treatment marked with (*)

*Δ*_ave_	*σ*_PL_	*λ*_ave_	*σ*_*λ*_
58	56%* → 29%	794 nm* → 787 nm	3.31 nm* → 1.02 nm

An average PL enhancement of 58 fold is observed upon OA treatment. The standard deviation in PL intensity decreases from 56% to 29%. This demonstrates that the OA treatment both improves PL and spatial homogeneity of brightness. Spectral narrowing is also observed with an average blue shift *λ*_ave_ of 7 nm with improved spectral uniformity given by a 69% reduction in *σ*_*λ*_ from the untreated to the treated case. The median PL enhancement *Δ*_median_ was calculated to be ∼ 61 fold. The normalised median spectra show a blue shift of 12 nm (798 nm → 786 nm) in the spectral peak and reduction of 5.5 nm (27.2 nm → 21.7 nm) in the full width half maximum (FWHM) from the untreated to treated case. As also observed in OA treated WS_2_,^1^ the spectral blue-shift and line-width narrowing of MoSe_2_ PL may be attributed to changes in strain induced by ligand coordination. These results however establish the efficacy of the OA treatment to enhance the PL properties of MoSe_2_.

**Fig. 2:**
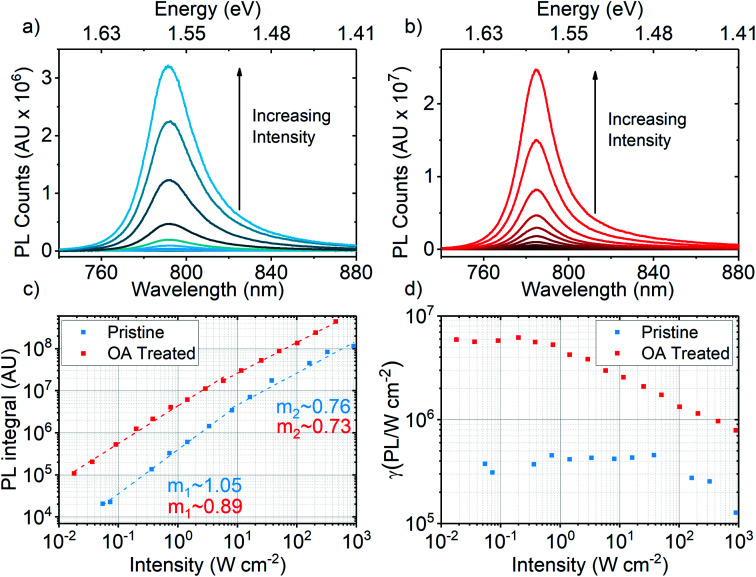
(a) and (b) Raw PL spectra from excitation intensity series of untreated and OA treated MoSe_2_, respectively; (c) excitation intensity series derived from PL integrals of spectra (a and b) for untreated (blue) and OA treated (red) monolayers; (d) ratio of PL integral to excitation intensity *i.e.* relative PLQE (*γ*) as a function of excitation intensity for untreated (blue) and OA treated (red) monolayers.

To probe the exciton dynamics that accompany the PL enhancement, we look at the excitation intensity dependent room temperature PL of a monolayer before and after OA treatment. Fig. 2a–d show the results derived from a room temperature steady state excitation intensity dependent PL series over five orders of magnitude. Intensities range between 0.018 W cm^−2^ and 909 W cm^−2^, remaining well below 9000 W cm^−2^ to avoid thermal damage.^16^

Fig. 2a shows no noticeable changes in spectral properties with increased excitation intensity in the untreated monolayer. When treated with OA, as shown in Fig. 2b, overall spectral narrowing compared to that of the untreated case was observed; however no additional spectral components are observed, unlike the case of OA treated WS_2_ which shows strong trion contribution at high excitation intensities.^1^

Fig. 2c shows a log–log plot of PL integrals as a function of excitation intensity for untreated (blue) and treated (red) samples. The gradients (*m*) of the series represent the exponent to the power law fit, *I* = *P*^*m*^.^33^ As such, the *m* values indicate the exciton recombination regimes observed. Fig. 2d shows the ratio of PL to excitation intensity (*γ*), which serves as a relative PLQE value. At low intensities, the untreated sample shows slight super-linear behaviour (*m*_1_ ∼ 1.05), which is indicative of some degree of exciton trapping^1^ between 0.06 W cm^−2^ and 0.8 W cm^−2^. This suggests a lack of non-radiative exciton–exciton annihilation, as given by the little variation in *γ* ratio values between 1 W cm^−2^ and 10 W cm^−2^, albeit with low PLQE. Beyond 10 W cm^−2^, the trend becomes sublinear (*m*_2_ ∼ 0.76), indicating the onset of non-radiative exciton–exciton annihilation.^16,20,33,34^ However, non-radiative trap assisted recombination processes dominate throughout the series, given the low PLQE of untreated TMD monolayers.^1,16,17,20,28–30,32–34^

When treated with OA, the emission follows a sub-linear power law exponent *m*_1_ of ∼ 0.89 even at lower power, signifying the immediate onset of non-radiative exciton–exciton annihilation, and becomes more drastic at higher excitation intensities where *m*_2_ is ∼ 0.73. These trends are reflected in the *γ* ratio which shows a general gradual reduction between 0.02 W cm^−2^ and 0.76 W cm^−2^ before sharply decreasing thereafter due to intensified exciton–exciton annihilation. The immediate exciton–exciton annihilation seen in the OA treated sample is consistent with trap-free exciton diffusion, similar to what has been observed with OA treated WS_2_.^1^ The increase in relative PLQE, *γ*, by an average factor of ∼17 between 0.02 and 0.1 W cm^−2^ also confirms significant reduction in non-radiative recombination *via* trap states.

**Fig. 3:**
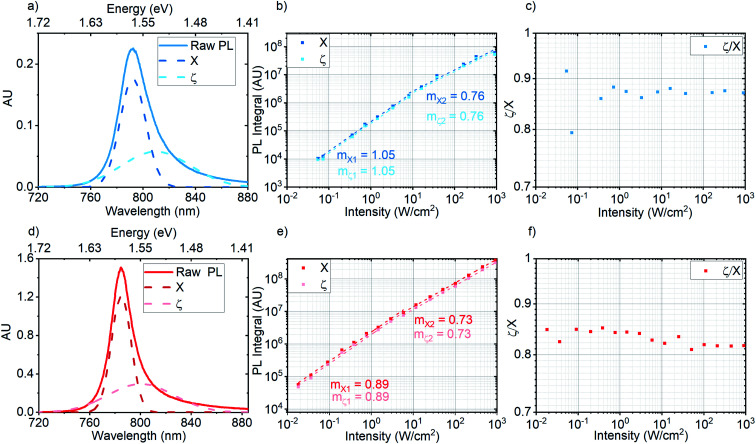
(a) Raw PL spectrum of pristine MoSe_2_ (blue) taken in the high intensity regime (327 W cm^−2^). Dashed dark blue and light blue Gaussian fits represent the neutral exciton (*X*) and a low energy species (*ζ*), respectively; (b) excitation intensity series of neutral exciton (*X*) and low energy species (*ζ*) in pristine MoSe_2_; (c) ratio of *ζ* to *X* as a function of excitation intensity in pristine MoSe_2_; (d) raw PL spectrum of OA treated MoSe_2_ (red) taken in the high intensity regime (455 W cm^−2^). Dashed maroon and pink Gaussian fits represent the neutral exciton (*X*) and a low energy species (*ζ*), respectively; (e) excitation intensity series of neutral exciton (*X*) and low energy species (*ζ*) in OA treated MoSe_2_; (f) ratio of *ζ* to *X* as a function of excitation intensity in OA treated MoSe_2_.

We attempt to characterize the exciton species that contribute to PL of pristine and OA treated MoSe_2_. Fig. 3a–f show the results obtained from deconvoluting each PL spectrum in the pristine case (Fig. 3a–c) and corresponding OA treated case (Fig. 3d–f). As per previous studies,^1,42,43^ Gaussian fits (shown in ESI Fig. 3 and 4†) were used to identify the emissive excitonic species in the spectra. Fig. 3a and d show the raw PL spectra of pristine (blue) and OA treated (red) samples taken at 327 W cm^−2^ and 455 W cm^−2^, respectively, which lies within the excitation intensity regime for trion emission in OA treated WS_2_.^1^ The Gaussian fits *X* and *ζ* represent neutral exciton and a low energy spectral component, respectively. Fig. 3b shows the excitation intensity series for *X* and *ζ* in pristine MoSe_2_. We observe that both components exhibit the same power law exponents (*m*) and (thus) recombination dynamics seen in the ensemble PL excitation intensity series of pristine MoSe_2_, as shown in Fig. 2c (blue series). Similarly, for the OA treated case shown in Fig. 3e, *X* and *ζ* obey the same recombination dynamics seen in the ensemble PL excitation intensity series in Fig. 2c (red line). Fig. 3c and e show the ratio of *ζ* to *X* as a function of excitation intensity for pristine and OA treated MoSe_2_, respectively. In both cases, *ζ*/*X* is fairly constant and remains below unity throughout the series. On average, the *ζ*/*X* ratio in the OA sample is slightly lower than in the pristine sample. This indicates a slight increase in the proportion of neutral excitons in the OA case compared to that in the pristine case.

The constant *ζ*/*X* < 1 ratio in both cases indicates the dominance of neutral excitons compared to low energy species such as trions^44^ throughout the series. Trions in particular, evolve from the binding of neutral excitons to free photoionized charges and have been characterized in room temperature WS_2_ PL measurements, which show the growth of a broad and red-shifted low energy feature as a function of increasing excitation intensity.^1,42–44^ While strong neutral exciton contributions are observed throughout the series, easily discernible trion evolution is not apparent in both pristine and OA treated MoSe_2_ PL spectra. A recent study on exciton and trion dynamics in MoSe_2_ concluded that trion formation is suppressed at room temperature due to changes in localisation effects.^45^ To this end, OA treatment simply improves neutral exciton PL by reducing the density of non-radiative channels which may take the form of trap states caused by chalcogen vacancies. As per the work cited,^45^ identifying the effects of OA treatment on trion emission in MoSe_2_ would require low temperature PL studies.

**Fig. 4:**
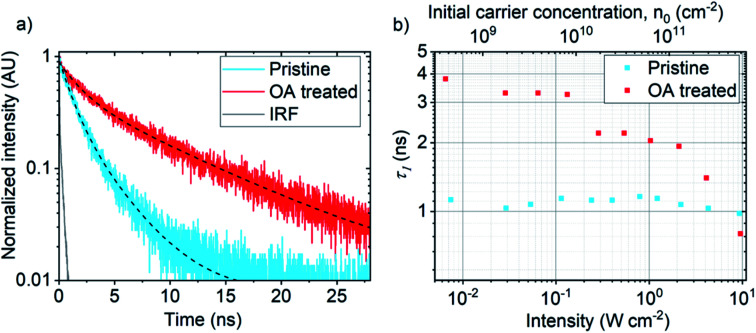
(a) Time resolved PL signals of pristine (blue) and OA treated (red) MoSe_2_ monolayers with bi-exponential decay fits (black dashed lines) measured at comparable pump intensities; 0.054 W cm^−2^ (pristine sample) and 0.064 W cm^−2^ (OA sample) using 550 nm, 5 MHz pulsed excitation. (b) Variation of fast PL decay component, *τ*_1_ as a function of initial carrier concentration, *n*_0_ and pump intensities (W cm^−2^).

To gain further insights into the exciton dynamics present in OA treated MoSe_2_, we employ time-resolved photoluminescence (TRPL) microscopy. Fig. 4a shows normalized PL decay signals at room temperature under comparable low intensity 550 nm, 5 MHz pulsed laser excitation. Signals were detected using a visible range single photon avalanche diode (VIS-SPAD) *via* 750 nm long pass and 900 nm short pass filters, removing laser excitation and collecting MoSe_2_ PL only. Pulsed excitation intensities used were 0.054 W cm^−2^ and 0.064 W cm^−2^ for pristine (blue) and OA treated (red) cases, respectively. Both signals are best described by a bi-exponential decay model (black dashed lines) consisting of fast *τ*_1_ and slow *τ*_2_ components. The pristine sample (blue) exhibits PL decays with *τ*_1_ of ∼ 1.07 ns and *τ*_2_ of ∼ 3.06 ns. For the OA treated case (red), PL lifetimes are extended by a factor of 3× and 3.8× *vs.* the pristine sample for fast and slow decays, respectively, with *τ*_1_ of ∼ 3.3 ns and *τ*_2_ of ∼ 12.07 ns. The overall increase in PL lifetimes due to OA treatment *vs.* the pristine case reveals a suppression of non-radiative decay channels.

Fig. 4b shows the variation in the fast decay component *τ*_1_ as a function of initial carrier concentration *n*_0_ over four orders of magnitude. Initial carrier concentrations were computed using openly available MoSe_2_ steady state absorption data.^28^ The excitation intensities used fall within the range used for the steady state excitation series shown in Fig. 2c and d. As shown in ESI Fig. 5,† all decays in the series fit a bi-exponential model. The pristine case shows very little variation in *τ*_1_ over the range of *n*_0_ which indicates exciton trapping. In contrast, OA treated MoSe_2_ shows a general reduction in *τ*_1_ in the *n*_0_ range measured. This lies in agreement with the sub-linear trend measured within the same excitation intensity regime shown in Fig. 2c, which indicates the immediate onset of exciton–exciton annihilation at low excitation fluences. Accordingly, the observed reduction in *τ*_1_ as a function of *n*_0_ in the OA treated sample implies non-trap limited movement of excitons and thus provides further evidence for trap state passivation due to OA treatment. ESI Fig. 6† shows the equivalent comparison for the slow decay component, *τ*_2_, for which the pristine case shows a general increase in lifetime as a function of *n*_0_ in accordance with trap state filling while the OA treated sample shows a reduction in lifetime as a function of *n*_0_.

In summary, steady state PL measurements presented so far show that OA treatment greatly enhances the PL of monolayer MoSe_2_ and optical quality in terms of emission linewidth and spatial homogeneity of brightness. Steady state excitation intensity dependent PL and TRPL studies reveal trap-free neutral exciton movement in OA treated MoSe_2_. The observed enhanced PL and trap free exciton annihilation dynamics together support the hypothesis of true defect passivation by OA. In ESI Fig. 7,† we include the PL spectra of pristine and OA treated WSe_2_ from a single spot on the monolayer to emphasize the versatility of OA treatment.

The exact surface chemistry that gives rise to the observed optical improvement is not fully clear at the moment and future experimental and theoretical studies will be required to understand the underlying mechanism. We note that the Raman spectra of pristine and OA treated MoSe_2_ in ESI Fig. 8† show no distinguishable structural changes. We however consider that the treatment mechanism would be linked to passivation of chalcogen defects through oleate coordination to Mo dangling bonds. Chemical passivation of these vacancy sites suppresses excitonic trap states, resulting in vastly improved PL efficiency, due to direct band-edge recombination and trap-free exciton movement. In addition, the formation of an OA layer with bulky alkyl chains may provide an insulating encapsulant to the TMD monolayer analogous to hBN encapsulation, resulting in better protection from reactive species formed from atmospheric oxygen and water, and external trap states outside the monolayer from adsorbants, thereby contributing to the observed improvements in optical characteristics. To corroborate the oleate-defect coordination mechanism, we suggest further experiments using an analogous 18 carbon molecule such as *cis*-9-octadecene.

**Fig. 5:**
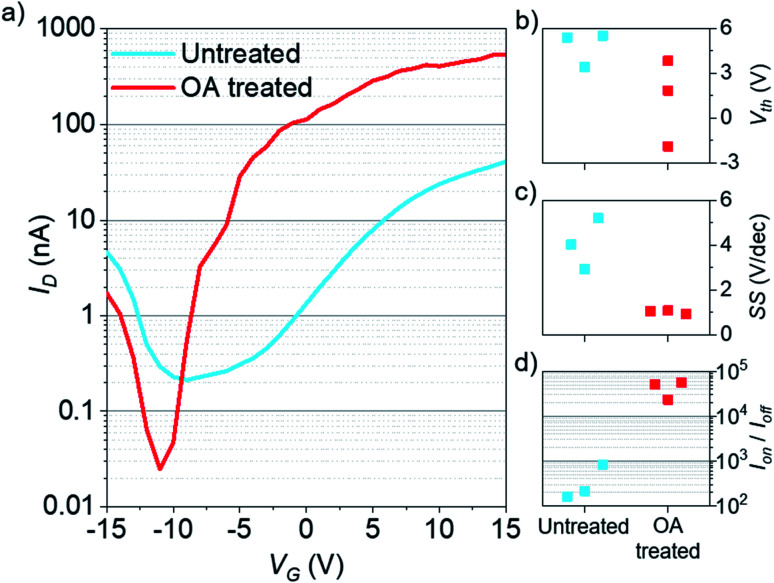
(a) Transfer characteristics of the same back-gated monolayer MoSe_2_ field effect transistor before (blue) and after (red) OA treatment. (b) Threshold voltage, (c) subthreshold swing, (d) on–off current ratio for three MoSe_2_ transistors before (blue) and after (red) OA treatment.

Finally, to assess the impact of OA treatment on the electronic properties of monolayer MoSe_2_, we test back-gated field effect transistors. Fig. 5a shows the predominantly n-type transfer characteristics of MoSe_2_ before OA passivation, consistent with previous reports.^46^ The as-fabricated devices are then treated with OA under the same conditions as described in the Experimental section. After OA treatment, n-type transfer characteristics are preserved. There is a relatively small threshold voltage (*V*_th_) shift from *V*_th,Un_ = 4.8 ± 1 V to *V*_th,OA_ = 1.3 ± 2.3 V (Fig. 5b), indicating no substantial change in doping induced by the OA treatment. After OA treatment, the devices consistently show an improved subthreshold swing (SS) from SS_Un_ = 4 ± 0.9 V dec^−1^ to SS_OA_ = 1 ± 0.1 V dec^−1^ (Fig. 5c), which indicates a reduction in interface charge trap density and is consistent with the notion of defect passivation by OA. A higher on-state current, due to reduced charge trapping, and larger off-state resistance after OA treatment lead to an improved on–off current ratio up to ∼5 × 10^4^ (Fig. 5d).

## Conclusions

In conclusion, we have established OA surface treatment of MoSe_2_ as an effective means of achieving drastically improved PL yields and trap free PL dynamics, as compared with untreated monolayers. PL statistics reveal that OA treatment yields monolayers of improved optical quality by way of bright spatially homogeneous PL with a narrow spectral linewidth. A steady state excitation intensity dependent PL series reveals significantly improved ‘PLQE’ with trap-free exciton dynamics, which is taken as initial evidence of passivation of non-radiative trap states by OA ligands. Analysis of the excitonic species present in the excitation intensity series verifies dominant neutral exciton recombination in OA treated MoSe_2_ under low to high excitation intensities. Consistent with improved steady state PL, time resolved PL studies reveal significantly improved PL lifetimes. The reduction in PL lifetimes as a function of initial carrier concentration also indicates trap free exciton movement, which further supports the hypothesis of PL enhancement as a result of ligand passivation. By way of surface chemical interactions between OA and monolayer MoSe_2_, we hypothesise that the OA ligands coordinate to Mo dangling bonds at Se vacancies, which are known to be exciton trap states, thus passivating them and yielding increased radiative efficiency. The insulating ligands may also protect the monolayer from atmosphere induced doping and surface induced strain, thus acting as an encapsulant, which may also contribute to the PL linewidth narrowing. OA treated MoSe_2_ based FETs show no significant additional doping. However, we observe a considerable improvement in subthreshold swing with orders of magnitude increase in the on–off ratio, which provides further evidence of trap or defect passivation by OA. In essence, the results show that OA treatment is an effective, simple and versatile ‘wet’ chemistry technique than can improve the PL characteristics of a selenide based TMD. Combined with previous studies on sulphur based TMDs, these results establish the ‘ligand’ based passivation approach as a universal defect treatment protocol for both sulphide and selenide based TMDs.

## Author contributions

A. O. A. T. prepared samples, performed PL and TRPL measurements, analysed data, wrote manuscript and produced TOC images; J. A.-W. and Y. F. performed transistor preparation, electrical characterization and electronic transport data interpretation; N. G. assembled the TRPL setup; J. X. provided insights into surface chemistry; R. P. assisted in PL data analysis; Z. L. assisted in TOC image preparation.

## Conflicts of interest

There are no conflicts of interest to declare.

## Supplementary Material

NA-003-D0NA01014F-s001
