# Supplementary material for: Giant photoluminescence enhancement in MoSe2 monolayers treated with oleic acid ligands
Source: Nanoscale Adv. 2021 Jun 12;3(14):4216–25. doi: 10.1039/d0na01014f (PMC8276785; doi:10.1039/d0na01014f)
Supplement: NA-003-D0NA01014F-s001 [file NA-003-D0NA01014F-s001.pdf]

Electronic Supplementary Material (ESI) for Nanoscale Advances.
This journal is © The Royal Society of Chemistry 2021

Supporting Information

Giant Photoluminescence Enhancement in MoSe2 Monolayers treated with Oleic Acid Ligands

Arelo O.A Tanoh[1,2], Jack Alexander-Webber[3], Ye Fan[3], Nicholas Gauriot[1], James Xiao[1], Raj Pandya[1], Zhaojun Li[1], Stephan Hofmann[3], Akshay Rao[1]*

[1]Cavendish Laboratory, Cambridge, JJ Thomson Avenue, CB3 0HE, Cambridge, United Kingdom

[2]Cambridge Graphene Centre, University of Cambridge, 9 JJ Thomson Avenue, Cambridge, CB3 0FA, Cambridge, United Kingdom

[3]Department of Engineering, University of Cambridge, JJ Thomson Avenue, CB3 0FA Cambridge, United Kingdom

*E-mail: ar525@cam.ac.uk

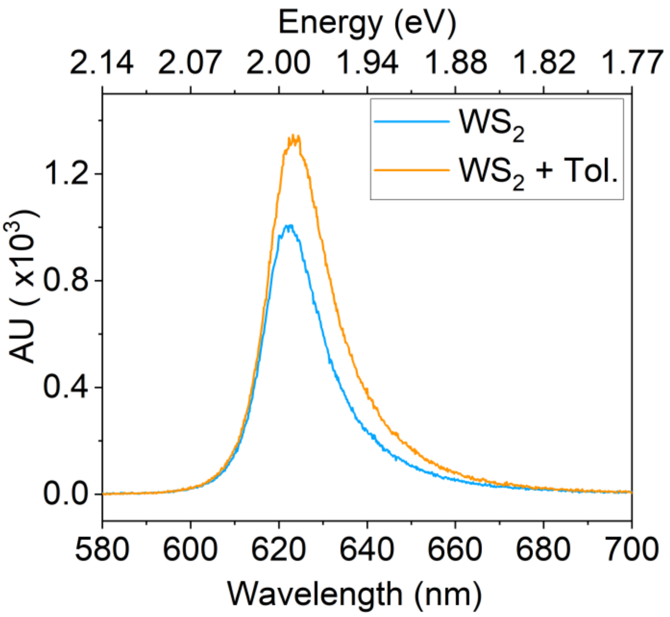

SI Figure 1: Effect of Toluene on WS_2 PL

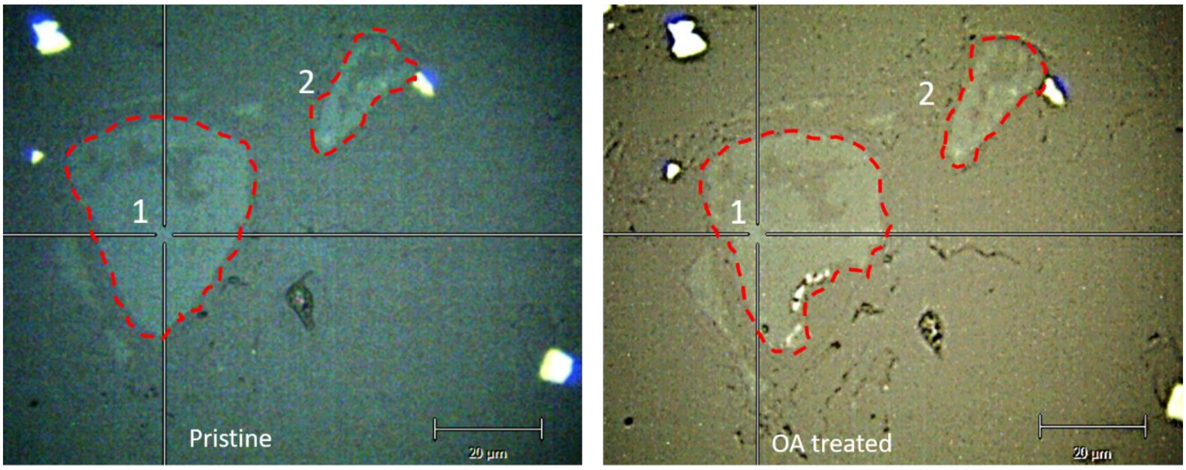

SI Figure 2: Optical micrographs of $MoSe_2$ monolayers before (LHS) and after (RHS) OA treatment. Scalebar represents 20 μm. Slight delamination in monolayer 1 attributed to surface tension effects of OA.

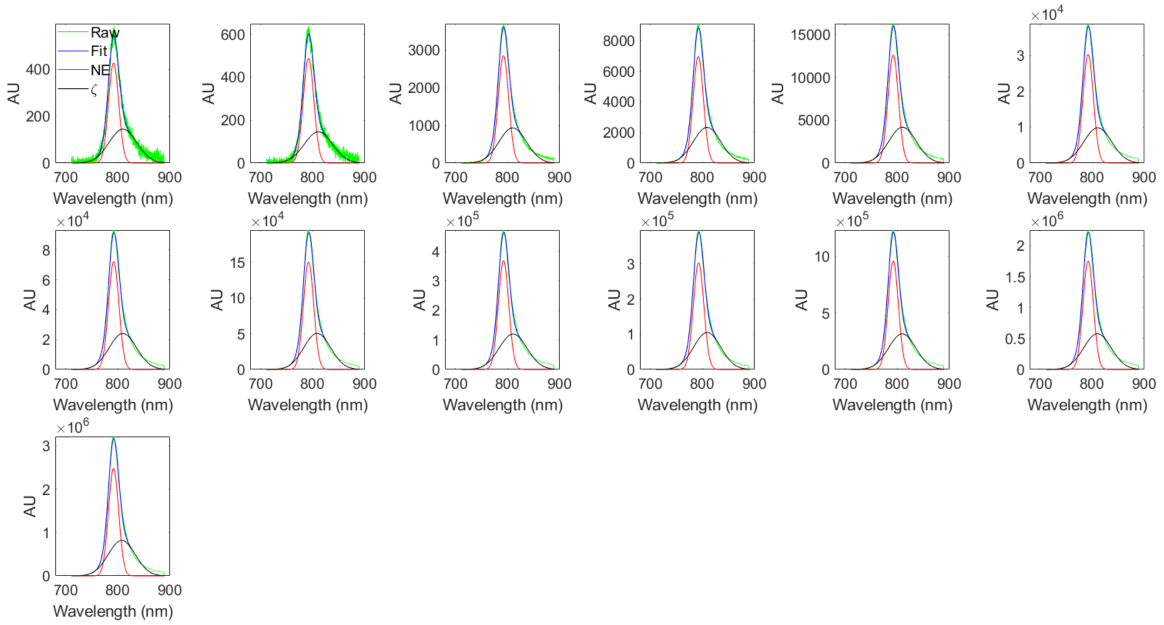

SI Figure 3: Gaussian fits of pristine $MoSe_2$ monolayer PL spectra

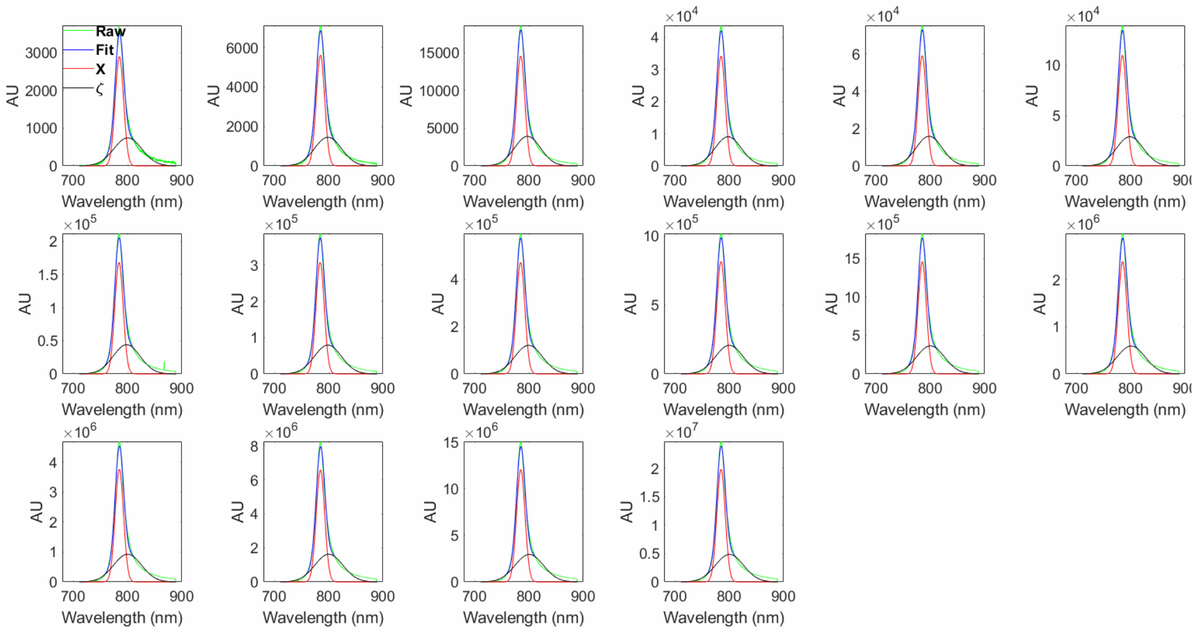

SI Figure 4: Gaussian fits of OA treated $MoSe_2$ monolayer PL spectra

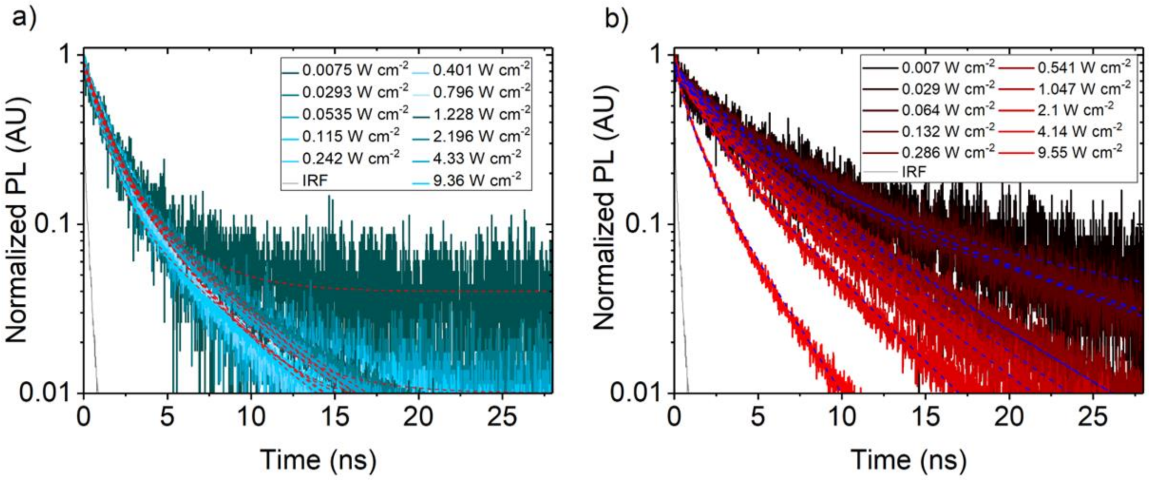

<u>SI Figure 5 a-b</u>: Time resolved photoluminescence signals for pristine (blue) and OA treated (red) samples with bi-exponential decay fits (red dashed line in pristine spectra and blue dashed lined in OA treated spectra).

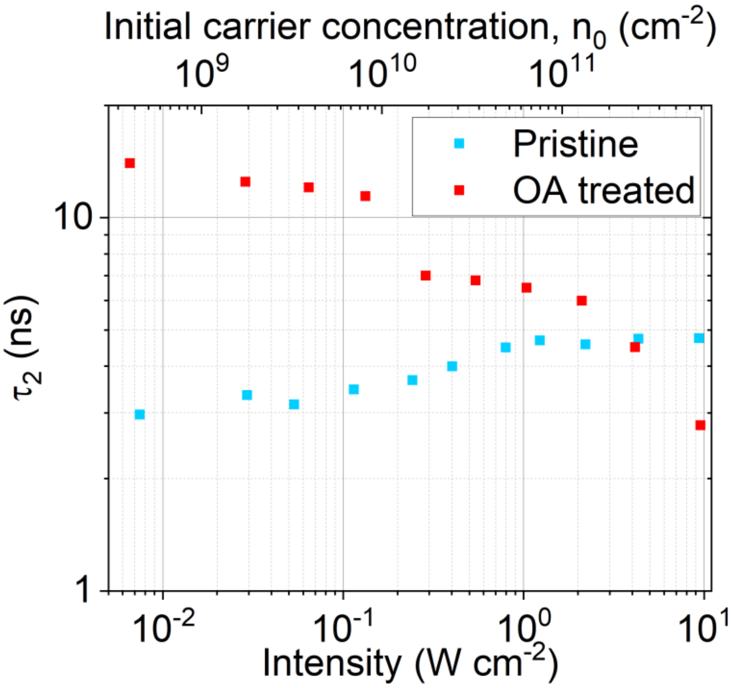

<u>SI Figure 6:</u> Variation of slow decay component, τ_2, of pristine (blue) and OA treated (red) $MoSe_2$ time resolved PL signals with initial carrier concentration and pump intensities (W cm^{-2}).

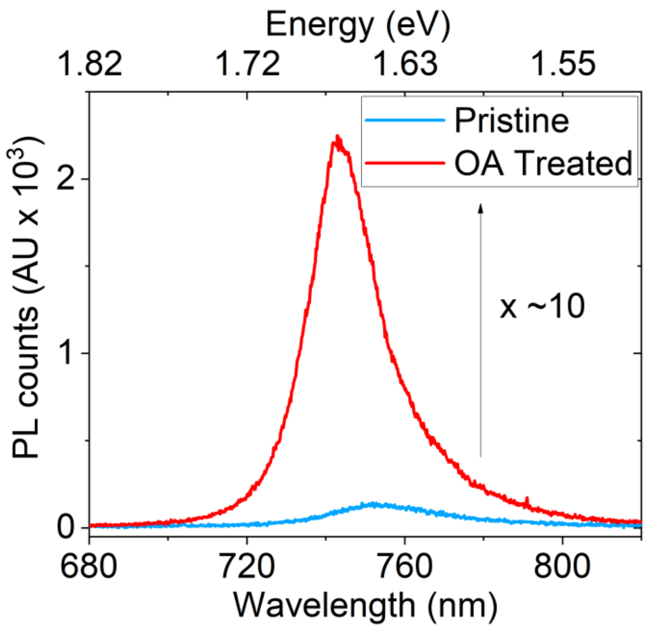

SI Figure 7: Raw PL spectra of a single spot on a WSe_2 monolayer on Si-SiO_2 (90 nm) treated with OA.

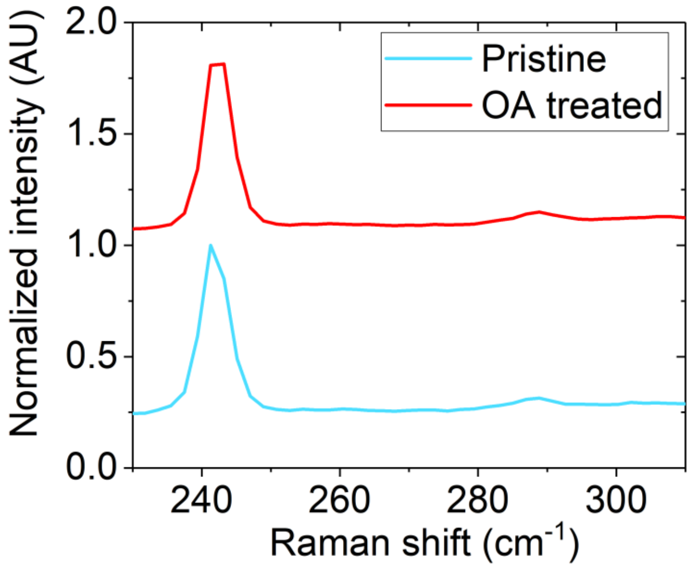

SI Figure 8: Raman spectra of pristine (blue) and OA treated $MoSe_2$ monolayers on glass substrate.
